# Geometrical Optics Restricted Eavesdropping Analysis of Satellite-to-Satellite Secret Key Distillation

**DOI:** 10.3390/e23080950

**Published:** 2021-07-25

**Authors:** Ziwen Pan, Ivan B. Djordjevic

**Affiliations:** Department of Electrical & Computer Engineering, College of Engineering, The University of Arizona, 1230 E Speedway Blvd, Tucson, AZ 85721, USA; ivan@arizona.edu

**Keywords:** geometrical optics restricted eavesdropping, secret key distillation, satellite-to-satellite

## Abstract

Traditionally, the study of quantum key distribution (QKD) assumes an omnipotent eavesdropper that is only limited by the laws of physics. However, this is not the case for specific application scenarios such as the QKD over a free-space link. In this invited paper, we introduce the geometrical optics restricted eavesdropping model for secret key distillation security analysis and apply to a few scenarios common in satellite-to-satellite applications.

## 1. Introduction

Quantum key distribution is known to guarantee unconditional security. The first QKD protocol, BB84, was developed in 1984 [1], which uses the polarization states of single photons to safely distribute keys. This was also known as the first discrete variable (DV)- QKD. Different protocols have since been studied, such as device-independent protocols that study the security with compromised apparatus [2,3,4,5], high dimensional protocols that exploit high dimensional degrees of freedom to increase the key rate [6,7,8,9,10] and decoy state protocols [11,12,13] that use decoy states against the photon-number-splitting attack [14]. Another major category in the study of QKD protocols, the continuous variable (CV) protocols [15,16] that encode keys into CV observables of carrier fields [17], are known to be more easily implementable for their compatibility with current communication devices instead of relying on single-photon generation and detection like most DV protocols.

Generally, in this paper, we assume that Alice uses a multi-photon source governed by the mean photon number without photon-number-resolving detectors so that she is limited in knowing whether she is transmitting a multi-photon wave packet, for example, if she only has a Geiger mode detector that clicks when one or more photons are detected. For security analysis of the quantum key distribution under these assumptions, conventionally, an omnipotent eavesdropper (Eve) that can gather information from the multi-photon wave packets transmitted from Alice to Bob by collecting every photon that does not arrive at Bob’s receiver is assumed [18,19,20,21,22,23,24,25]. However, this is not the case for some specific application scenarios. For example, it would be reasonable to assume that the eavesdropper’s (Eve’s) power collection ability is limited due to the size of her aperture in an optical wireless channel from Alice to Bob. In [26,27], geometrical optics restricted eavesdropping analysis was proposed, considering the reasonably limited power collection ability of Eve. In [28,29,30,31,32,33], some of the applications of this restricted Eve model were introduced.

In this invited paper, we present some of the applications of the geometrical optics restricted model. In Section 2, we briefly introduce the power-collection-restricted eavesdropping model and give the lower and upper bound expressions. In Section 3.1, we showcase geometrical optics restricted eavesdropping analysis with a case where the eavesdropper has an aperture of a limited size in the same plane as Bob’s while investigating the exclusion zone as one of Bob’s defense strategies. In Section 3.2, we further assume that Eve’s aperture can be dynamically positioned and provide the results while optimizing this eavesdropping strategy. We conclude that the geometrical optics restricted eavesdropping model is suitable for multiple application scenario analysis.

## 2. Geometrical Optics Restricted Eavesdropping Model

**Figure 1:**
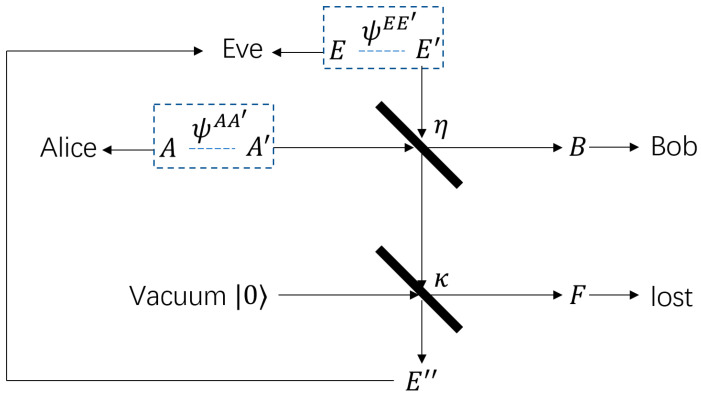
Geometrical optics restricted model wiretap channel notation [26].

As is illustrated in Figure 1, instead of assuming that Eve collects all the photons outside of Bob’s receiver, only a fraction κ of them is collectable by Eve, denoted here as a wiretap channel with a κ-transmissivity beamsplitter. Here, η is the Alice-to-Bob channel transmissivity, μ is the input mean photon number per mode on Alice’s side, and $n_{e}$ is the noise mean photon number per mode on Eve’s side. $\psi^{AA^{\prime }}$ and $\psi^{EE^{\prime }}$ in Figure 1 are entanglement pairs. Alice would keep mode A and send mode $A^{\prime }$ to Bob, and in the most general case, Eve would also use entanglement pairs to eavesdrop, retaining mode E and sending mode $E^{\prime }$ into the channel. In [26], the lower bound on the achievable key rate for direct and reverse reconciliation is shown below:

(1) $$K_{\to }\geq \beta g\left(n_{e}\left(1-\eta \right)+\eta \mu \right)-\sum_{i}g\left(\frac{\nu_{y_{i}}^{ER}-1}{2}\right)-\beta g\left(n_{e}\left(1-\eta \right)\right)+g\left(n_{e}\left(1-\eta \kappa \right)\right),$$

(2) $$K_{\leftarrow }\geq \beta g\left(\mu \right)-\underset{i}{\sum }g\left(\frac{\nu_{y_{i}}^{ER}-1}{2}\right)-\beta g\left(\mu -\frac{\eta \mu \left(1+\mu \right)}{1+n_{e}-n_{e}\eta +\eta \mu }\right)+\underset{i}{\sum }g\left(\frac{\nu_{y_{i}}^{ER}-1}{2}\right)\;,$$

(3) $$g\left(x\right)=\left(x+1\right)\log_{2}\left(x+1\right)-x\log_{2}x$$

with detailed expressions of $\nu_{y_{i}}^{ER}$ available in [26]. Here, β is the reconciliation efficiency, which is set to β=1 throughout this paper.

The upper bound in a pure loss channel ($n_{e}=0$) is shown to be [26]

(4) $$K\leq \log_{2}\frac{\eta +\kappa \left(1-\eta \right)}{\kappa \left(1-\eta \right)}\;,$$

while the upper bound in a thermal noise channel does not have a closed form expression. Detailed calculations can be found in Appendix A of [26].

## 3. Applications on Satellite-to-Satellite Secret Key Distillation

In this section, we study some applications of the geometrical optics restricted model analysis that would be common in satellite-to-satellite links where Eve’s collecting ability would be naturally limited due to the radius of her receiver aperture, which usually ranges from centimeters to decimeters for traditional free-space communication. If we take existing space applications into account for an upper-bounding estimation of Eve’s aperture size, the Giant Magellan Telescope, one of the largest optical observatories, has a primary mirror of a 12.5-m radius [34]. Other known aperture sizes of satellite-based applications are much smaller, such as the 1.2-m-radius primary mirror for the Hubble Space Telescope [35] and the 20-cm-radius aperture for NASA’s “Wide-field Infrared Survey Explorer” infrared telescope [36].

We analyze both the communication parties’ and Eve’s strategy by starting with a defense strategy from Bob’s side called an exclusion zone, under the aforementioned assumptions and considering the case where Eve’s aperture is in the same plane with Bob’s in Section 3.1. Then, in Section 3.2, we move forward from that and assume that Eve’s aperture can be dynamically positioned, concluding Eve’s strategy for eavesdropping. In this section, we assume that a Gaussian beam with a beam waist $W_{0}$ and wavelength λ=1550 nm is transmitted. The space temperature is set to T=3 K, and we calculate the noise mean photon number using the black body radiation equation:

(5) $$n_{e}=\frac{1}{e^{\frac{hf}{kT}}-1}\;,$$

where h is the Planck constant, f is the transmission center frequency, and k is the Boltzmann constant. We then calculate the power transmitted by Alice $P_{Alice}$, the power received by Bob $P_{Bob}$, the power received by Eve $P_{Eve}$, and the channel transmissivity η, and the restriction factor on Eve κ can be expressed as

(6) $$\eta =\frac{P_{Bob}}{P_{Alice}}\;,$$

(7) $$\kappa =\frac{P_{Eve}}{P_{total}\left(1-\eta \right)}\;,$$

In this section, we calculate the lower bound as the maximum of the direct reconciliation lower bound and the reverse reconciliation lower bound.

### 3.1. Bob’s Defense Strategy: Exclusion Zone

**Figure 2:**
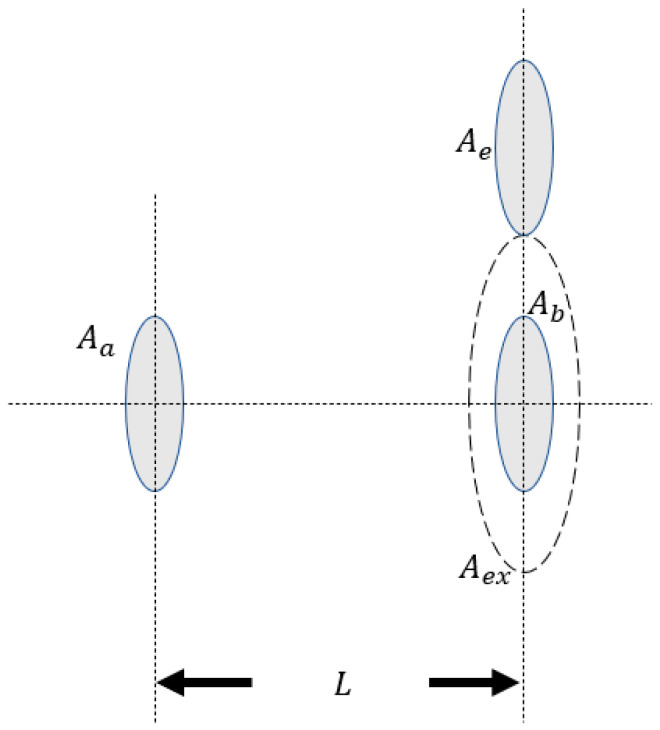
Limited size aperture of Eve in the same plane as Bob’s. Here, Bob is setting an exclusion zone around his receiver as a defense strategy.

In this subsection, we introduce the problem set-up of one of the most straightforward defense strategies of the communication parities: the so-called exclusion zone. In principle, the closer Eve is to the beam transmission axis from Alice to Bob, the more likely the legitimate communication parities would detect the eavesdropper’s presence (e.g., with a naïve approach such as a visible or infrared telescope or even radar to detect the eavesdropper’s presence and abort communication if a possible eavesdropper is detected within a certain range to the communication parities). In free-space channels such as the satellite links, it is also possible for Bob to have opaque material around his receiver to absorb any photons that might have arrived outside of his receiver’s aperture, preventing them from further propagation and possibly ending up in Eve’s receiver aperture. As is illustrated in Figure 2, the exclusion zone is denoted with a dashed circle around Bob’s receiver, excluding potential eavesdroppers to collect photons that arrive in this region. By definition, Bob’s aperture area is also part of the exclusion zone, since the photons arriving at Bob’s aperture would not be collectable by Eve. Here, more specifically, we say that Bob is setting up an exclusion zone if the area of the exclusion zone ($A_{ex}$) is larger than his receiver aperture area ($A_{Bob}$ or $A_{b}$). Other specified parameters include L being the transmission distance and $A_{Alice}$ ($A_{a}$) and $A_{Eve}$ ($A_{e}$) being the area of Alice’s aperture (radius $r_{a}$) and Eve’s aperture (radius $r_{e}$), respectively. The radii of Bob’s aperture and the exclusion zone are denoted as $r_{b}$ and $r_{ex}$ ($r_{ex}\geq r_{b}$). Here, the limited size of Eve’s aperture is placed in the same plane as Bob’s, since that would be the worst-case scenario for the purpose of our study under this exclusion zone assumption if Eve is not allowed between the Alice-to-Bob line of sight.

**Figure 3:**
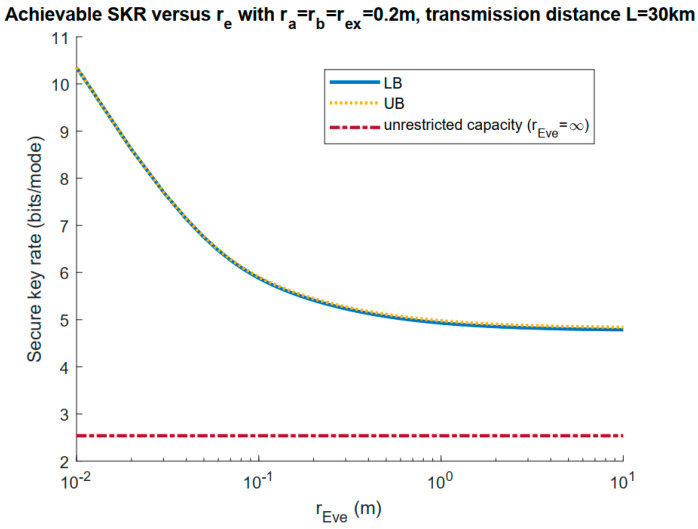
Achievable secure key rate lower and upper bound as functions of Eve’s aperture radius $r_{e}$, with $r_{ex}=r_{b}$. The unrestricted case (infinite-sized aperture on Eve’s side) is also included. Here, $W_{0}=r_{a}=r_{b}=r_{ex}=20$ cm.

To start with, we set $r_{ex}=r_{b}$ (no additional exclusion zone) and investigate how Eve’s aperture size would affect the achievable secure key rate lower bound (LB) and upper bound (UB), as shown in Figure 3. Here, we can see that under these parameters, the lower bound was quite close to the upper bound, which gave us the capacity in this scenario. As Eve’s aperture size increased, the achievable rate went down and saturated but still outperformed the unrestricted case capacity. The reason for this convergence is that the transmitted beam intensity was the strongest at its center and weakened fast in the outer regions. As such, up to some point, increasing Eve’s aperture size would only be able to gather photons from the regions far away from the beam center, thus making it ineffective in increasing Eve’s advantage. As a result of that, in the figure below, we only set Eve’s aperture radius to be 10 cm, equal to $r_{a}$ and $r_{b}$, for a fair comparison.

**Figure 4:**
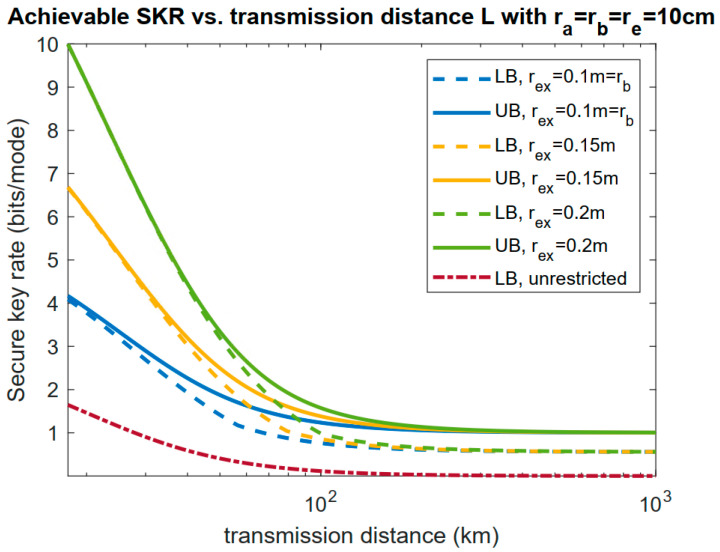
Achievable secure key rate lower and upper bounds as functions of the transmission distance. The unrestricted case (infinite size aperture on Eve’s side with $r_{ex}=r_{b}$) is also included. Here, $W_{0}=r_{a}=r_{b}=r_{e}=10$ cm.

In Figure 4, we set the exclusion zone radius to be $r_{ex}=15$ cm and 20 cm to compare the achievable rate lower bounds (LB) and upper bounds (UB) for the case without an additional exclusion zone. Here, we can see that with an aperture of a limited size on Eve’s side, the achievable secure key rate outperformed that of the unrestricted case. The lower bound and upper bound were quite close, which gave the range for the capacity. We can also see that an exclusion zone helped increase the key rate when the transmission distance was not too large. However, when the transmission distance was sufficiently large, the lower and upper bounds became constant, as proved in [30], when the collecting ability of Bob and Eve became proportional to their aperture sizes:

(8) $$\underset{L\to \infty }{\lim}\frac{P_{Eve}}{P_{Bob}}=\frac{A_{e}}{A_{b}}\;,$$

Here, we can see that an exclusion zone would not affect this saturation very much, as at a large transmission distance, the collecting ability of Bob and Eve became proportional to their aperture sizes as in Equation (8) when the area of an exclusion zone was not significantly larger than the receiver aperture sizes of Bob and Eve.

### 3.2. Eavesdropper’s Strategy: A Dynamically Positioned Aperture

**Figure 5:**
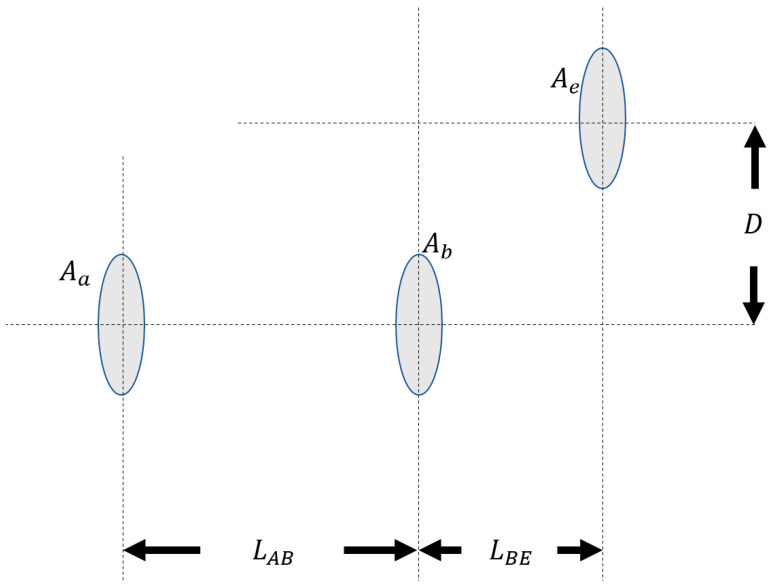
Eavesdropper dynamic positioning set-up.

In this subsection, we introduce and analyze one of the eavesdropper’s possible strategies with a dynamically positioned aperture, which would apply to the geometrical optics restricted model, where Eve could dynamically position her aperture behind Bob’s. As is illustrated in Figure 5, $A_{Alice}$($A_{a}$), $A_{Bob}$($A_{b}$), and $A_{Eve}$($A_{e}$) are the area of Alice’s aperture (radius $r_{a}$), Bob’s aperture (radius $r_{b}$), and Eve’s aperture (radius $r_{e}$), respectively. $L_{AB}$ is the distance between Alice’s and Bob’s aperture planes, while $L_{BE}$ is the distance between Bob’s and Eve’s aperture planes. D is the distance between Eve’s aperture center and the beam propagation line-of-sight path.

**Figure 6:**
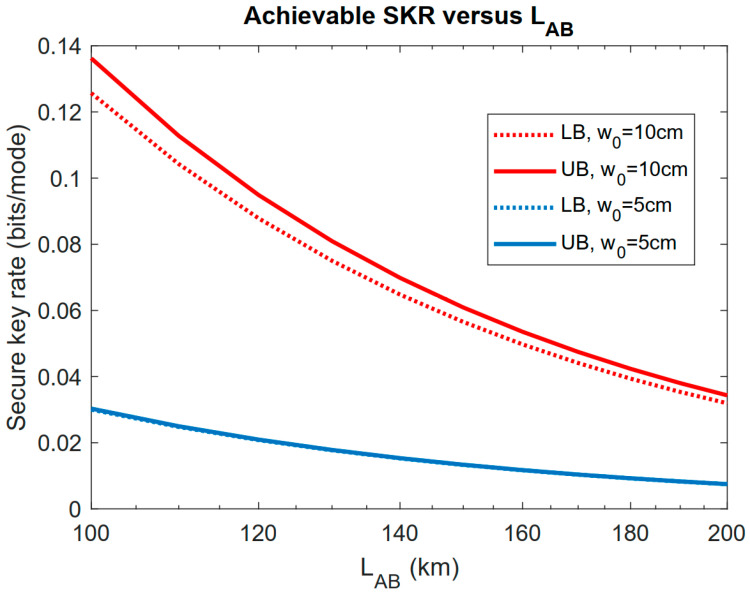
Lower and upper bounds of the achievable secure key rate versus $L_{AB}$ with $L_{BE}=L_{AB}$ and D=0. Bob’s and Eve’s aperture radii are $r_{b}=r_{e}=10$ cm.

As was proven in Equation (44) of [33], when $L_{AB}$ was sufficiently large, the optimal strategy for Eve was to set $L_{BE}=L_{AB}$ and D=0. Thus, we set $L_{BE}=L_{AB}$, D=0 and obtained the lower and upper bounds on the achievable secure key rate as in Figure 6. It is shown that in this case, the rate increased with the increase in $W_{0}$ as this decreased the divergence angle, making the beam more focused on Bob’s aperture plane. We can also see that Eve suppressed Alice and Bob’s achievable key rate compared with the similar distance range in Figure 4 by applying this strategy.

## 4. Discussion

In this invited paper, we briefly introduced the geometrical optics restricted model and presented a few cases applying this model to some common cases in free-space optical links such as the satellite-to-satellite channel. We showcased the achievable secure key rate lower and upper bounds and compared them to the unrestricted case. Furthermore, we investigated the strategy from both the communication parties’ side and Eve’s side within this model.
